# Integrated multi-omics profiling reveals a landscape of dramatic metabolic defect in *Artemisia annua*

**DOI:** 10.1093/hr/uhae174

**Published:** 2024-06-24

**Authors:** Wei Qin, Yongpeng Li, Hang Liu, Xin Yan, Xinyi Hu, Tiantian Chen, Saeed-ur Rahman, Junfeng Cao, Han Zheng, Ling Li, Kexuan Tang

**Affiliations:** College of Medical Technology, Chengdu University of Traditional Chinese Medicine, Chengdu 611137, China; Joint International Research Laboratory of Metabolic and Developmental Sciences, Key Laboratory of Urban Agriculture (South) Ministry of Agriculture, Plant Biotechnology Research Center, School of Agriculture and Biology, Shanghai Jiao Tong University, Shanghai 200240, China; Chongqing Key Laboratory of Sichuan-Chongqing Co-construction for Diagnosis and Treatment of Infectious Diseases Integrated Traditional Chinese and Western Medicine, Chengdu University of Traditional Chinese Medicine, Chengdu 611137, China; Joint International Research Laboratory of Metabolic and Developmental Sciences, Key Laboratory of Urban Agriculture (South) Ministry of Agriculture, Plant Biotechnology Research Center, School of Agriculture and Biology, Shanghai Jiao Tong University, Shanghai 200240, China; Joint International Research Laboratory of Metabolic and Developmental Sciences, Key Laboratory of Urban Agriculture (South) Ministry of Agriculture, Plant Biotechnology Research Center, School of Agriculture and Biology, Shanghai Jiao Tong University, Shanghai 200240, China; Joint International Research Laboratory of Metabolic and Developmental Sciences, Key Laboratory of Urban Agriculture (South) Ministry of Agriculture, Plant Biotechnology Research Center, School of Agriculture and Biology, Shanghai Jiao Tong University, Shanghai 200240, China; Joint International Research Laboratory of Metabolic and Developmental Sciences, Key Laboratory of Urban Agriculture (South) Ministry of Agriculture, Plant Biotechnology Research Center, School of Agriculture and Biology, Shanghai Jiao Tong University, Shanghai 200240, China; Joint International Research Laboratory of Metabolic and Developmental Sciences, Key Laboratory of Urban Agriculture (South) Ministry of Agriculture, Plant Biotechnology Research Center, School of Agriculture and Biology, Shanghai Jiao Tong University, Shanghai 200240, China; Joint International Research Laboratory of Metabolic and Developmental Sciences, Key Laboratory of Urban Agriculture (South) Ministry of Agriculture, Plant Biotechnology Research Center, School of Agriculture and Biology, Shanghai Jiao Tong University, Shanghai 200240, China; Joint International Research Laboratory of Metabolic and Developmental Sciences, Key Laboratory of Urban Agriculture (South) Ministry of Agriculture, Plant Biotechnology Research Center, School of Agriculture and Biology, Shanghai Jiao Tong University, Shanghai 200240, China; Joint International Research Laboratory of Metabolic and Developmental Sciences, Key Laboratory of Urban Agriculture (South) Ministry of Agriculture, Plant Biotechnology Research Center, School of Agriculture and Biology, Shanghai Jiao Tong University, Shanghai 200240, China; State Key Laboratory of Dao-di Herbs, National Resource Center for Chinese Materia Medica, China Academy of Chinese Medical Sciences, Beijing 100700, China; Joint International Research Laboratory of Metabolic and Developmental Sciences, Key Laboratory of Urban Agriculture (South) Ministry of Agriculture, Plant Biotechnology Research Center, School of Agriculture and Biology, Shanghai Jiao Tong University, Shanghai 200240, China; Joint International Research Laboratory of Metabolic and Developmental Sciences, Key Laboratory of Urban Agriculture (South) Ministry of Agriculture, Plant Biotechnology Research Center, School of Agriculture and Biology, Shanghai Jiao Tong University, Shanghai 200240, China

Dear Editor,

Trichomes are the specialized structures found on the surface of plants, categorized into glandular secretory trichomes (GSTs) and non-glandular trichomes based on their secondary metabolism capability [1]. *Artemisia annua* possesses both of the two types of trichomes, i.e., non-glandular T-shape trichomes (TSTs) and peltate GSTs, the latter being the primary site for the synthesis and accumulation of the specific antimalarial component, artemisinin [2]. Significant research efforts have been dedicated to elucidating the molecular mechanisms governing GST initiation and the metabolic pathways involved in artemisinin in *A. annua* [3, 4]. However, the comprehensive metabolism landscape of GSTs remains incompletely understood [5].

Here, we reported an *A. annua* mutant, which was accidentally discovered, exhibiting developmental defects in GSTs, named TRICHOME DEVELOPMENTAL DEFECTS 1 (*tdd1*) (Fig. 1a and b). Previous studies suggest that the GST cells are expected to possess denser cytoplasm indicative of secretory activity [6]. However, the cells of defective GSTs in *tdd1* were occupied by large vacuoles (Fig. 1c), revealing a compromised capacity for the secretion of secondary metabolites.

**Figure 1:**
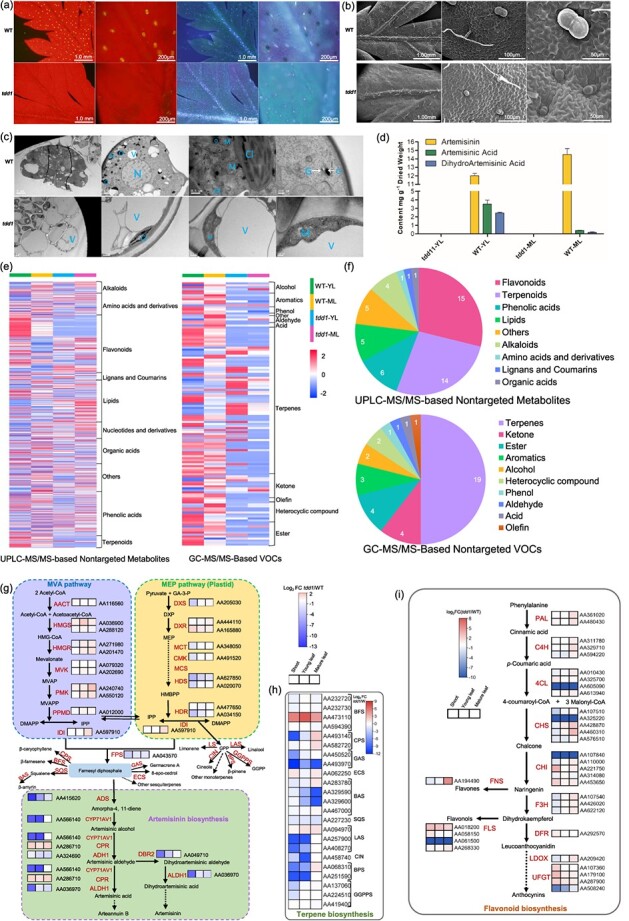
GSTs developmental defects in *Artemisia annua* lead to dramatic metabolic changes. **(a)** GSTs of WT and *tdd1*, images were taken under blue light or UV. **(b)** Scanning electron microscope (SEM) observations of GSTs. **(c)** Transmission electron microscopy (TEM) observation of GSTs. Cl, chloroplast; ER, endoplasmic reticulum; G, Golgi body; M, mitochondria; N, nucleus; O, osmiophilic material; OL, osmiophilic layer; V, vacuole. **(d)** The content of artemisinic acid, dihydroartemisinic acid and artemisinin (mg/g, Dried Weight, DW) in young leaves (YL) and mature leaves (ML) of WT and *tdd1*. Data are given as means ± SD (*n = 3*). **(e)** Overview of the nontargeted metabolites, and VOCs. The metabolite per row is Z-score standardized to −2 to 2. **(f)** Number of metabolites undetected in *tdd1*. Heatmap of genes involved in the artemisinin biosynthesis pathway **(g)**, other terpenes/terpenoids biosynthesis pathways **(h)**, and the flavonoid biosynthesis pathway **(i)**. The heatmaps represented log2 FC (*tdd1*/WT FPKM values). Each row of the heatmap represents one gene and each column represents one group. Abbreviations: The MVA pathway: AACT, Acetyl-CoA C-acetyltransferase; HMGS, Hydroxymethylglutaryl-CoA synthase; HMGR, 3-hydroxy-3-methylglutaryl CoA reductase; MVK, Mevalonate kinase; PMK, Phosphomevalonate kinase; PPMD, Diphosphomevalonate decarboxylase; IDI, Isopentenyl-diphosphate delta-isomerase; The MEP pathway: DXS, 1-deoxy-D-xylulose-5-phosphate synthase; DXR, 1-deoxy-D-xylulose-5-phosphate reductoisomerase; MCT, 2-methyl-D-erythritol-4-phosphate cytidylyltransferase; CMK, 4-(Cytidine 5′-diphospho)-2-C-methyl-D-erythritol kinase; MCS, 2-C-methyl-D-erythritol 2,4-cyclodiphosphate synthase; HDS, 4-hydroxy-3-methylbut-2-enyl diphosphate synthase; HDR, 4-hydroxy-3-methylbut-2-enyl-diphosphate reductase; The artemisinin biosynthesis pathway: FPS, farnesyl pyrophosphate synthase; ADS, Amorpha-4,11-diene synthase; CYP71AV1, cytochrome P450 dependent hydroxylase; ADH1, Alcohol dehydrogenase 1; CPR, cytochrome P450 oxidoreductase; ALDH1, aldehyde dehydrogenase 1; DBR2, double bond reductase 2; Other terpenes biosynthesis pathways: LAS, linalool synthase; LS, limonene synthase; CIN, cineole synthase; BPS, beta-pinene synthase, BFS, beta-farnesene synthase; CPS, beta-caryophyllene synthase; GAS, germacrene A synthase; ECS, 8-epi-cedrol synthase; SQS, squalene synthase; BAS, beta-amyrin synthase; The flavonoid biosynthesis pathway: PAL, phenylalanine ammonialyase; 4CL, coumarate-CoA ligase；CHS, chalcone synthase; C4H, cinnamate-4-hydroxylase; CHI, chalcone isomerase; FNS, flavone synthase; F3H, Flavanone 3-hydroxylase; FLS, flavonol synthase; DFR, fihydroflavonol 4-reductase; LDOX, leucoanthocyanidin dioxygenase; UFGT, UDP-glycose flavonoid glycosyltransferase.

Because artemisinin was primarily accumulated in GSTs, we determined the contents of artemisinic acid, dihydroartemisinic acid, and artemisinin, which are the key products in the artemisinin biosynthesis pathway, in young and mature leaves of *tdd1* and WT. Artemisinin, artemisinic acid and dihydroartemisinic acid were virtually undetectable in neither young nor mature leaves of *tdd1* (Fig. 1d). This result demonstrated that the mutation of GSTs can lead to the obstruction of the artemisinin metabolic pathway.

To further uncover the metabolic difference between *tdd1* and WT, young and mature leaves were collected for LC–MS based nontargeted metabolites analysis and GC–MS based volatile organic compounds (VOCs) analysis by MetWare (Wuhan, China) as described previously [7]. A total of 836 distinct nontargeted metabolites, classified into 10 classes, were detected (Fig. 1e). Among these, 52 metabolites were undetectable in both YL and ML of *tdd1*, primarily comprising to flavonoids (15) and terpenoids (14) (Fig. 1f). In the past decade, most studies have focused on the accumulation of artemisinin in GSTs, neglecting the potential effects of flavonoids [8]. Therefore, the nontargeted metabolome data can expand our understanding of the potential GST-specific flavonoids in *A. annua*. Accordingly, 131 VOCs including 11 classes were identified (Fig. 1e). There were 38 VOCs (mainly terpenes) undetected, in both YL and ML of *tdd1* (Fig. 1f). Apparently, according to our data, GST could be a specific site for the biosynthesis of many secondary metabolites, especially terpenes and flavonoids.

Multi-omics integration provides a comprehensive approach to elucidate the genetic and biochemical underpinnings of metabolism [9]. To get an insight into the transcriptional changes relevant to the metabolic defect of *tdd1*, we built transcriptomic profiles for shoot apical meristems, young leaves, and mature leaves of *tdd1* and WT. Given the substantial disparity in artemisinin accumulation between *tdd1* and WT, we deeply analysed the expression profile of enzymes in the artemisinin biosynthesis pathway. Notably, there was a certain difference in gene expression pattern within the MVA and MEP pathways between *tdd1* and WT (Fig. 1g). Specifically, most genes in the MVA pathway were slightly upregulated in *tdd1*, while most genes in the MEP pathway were downregulated, reflecting different metabolic fluxes related to GSTs defect. The GST-specific genes in the artemisinin biosynthesis pathway, including *ADS*, *CYP71AV*, *DBR2*, *ALDH1*, and *ADH1*, were also barely expressed in all tissues of *tdd1*, which precisely corresponded to the dramatic artemisinin accumulation block. Although the proportion of GSTs in the leaves is small, the GSTs defect still leads to changes in both MVA and MEP pathways. This further underscores the significance of GSTs in *A. annua*. The synthesis of volatile terpenes shows a strong correlation with the MEP pathway [10]. Therefore, the loss of the volatile terpenes in *tdd1* may lead to a metabolic inhibition in the MEP pathway, which corresponds to the down-regulation of the genes of the MEP pathway. Otherwise, the complex and variable metabolic crosstalk between the MEP and MVA pathways might cause the upregulation of the gene of MVA pathways. In brief, *tdd1* is an excellent mutant material to uncover the related mechanism.

To elucidate the variations in terpenes/terpenoids accumulation, we investigated the expression profile of the genes involved in the other terpenes/terpenoids biosynthesis pathways (Fig. 1h). Genes, including *CPS* (AA493140), *GAS* (AA450520, AA493970), *BAS* (AA329590, AA329600), *LAS* (AA257900, AA408270), *CIN* (AA458740), and *CPS* (AA068310, AA251590) showed dramatically low expression levels in *tdd1*. Integrated analysis of metabolomic and transcriptomic results suggested a substantial impediment in terpenes/terpenoids metabolism was largely hampered in *tdd1*. The genes with low expression levels, which exhibited the same pattern as the GST-specific genes in the artemisinin biosynthesis pathway, may play a crucial role in the synthesis and accumulation of GST-specific terpenes/terpenoids.

Since flavonoids constituted the majority of undetectable metabolites in *tdd1*, we further investigated the expression levels of the enzymes involved in flavonoid biosynthesis pathways. As a result, 33 DEGs were identified and changed in varying degrees between *tdd1* and WT (Fig. 1i). Notably, genes such as *4CL* (AA605090), *CHS* (AA325220), *CHI* (AA107840) and *FLS* (AA061500) exhibited extremely low expression levels in all samples from *tdd1*. It suggested a possible involvement of the correlated genes in the synthesis of the flavonoids that were absent in *tdd1*.

In summary, this study displays a systematical landscape of the transcriptional and metabolic changes between *tdd1* and WT, arising from the GSTs defect, and identifies specific genes that conduce to the disparate metabolites’ accumulation, thereby laying the foundation for future investigations on the contributions of these genes to the GSTs-specific terpenes/terpenoids and flavonoids biosynthesis.

## Data Availability

The original RNA-Seq data was deposited to the NCBI sequence read archive (SRA) database under the accession number PRJNA851562. All supplementary metabolomic and transcriptomic data is available in GitHub (https://github.com/Artemisiadata/Omics-data.git).
